# Attitudes towards interprofessional collaboration of Primary Care teams participating in the ‘More Doctors’ (Mais Médicos) program

**DOI:** 10.1590/1518-8345.2731.3018

**Published:** 2018-08-09

**Authors:** José Rodrigues Freire, Marcelo Viana da Costa, Carinne Magnago, Aldaísa Cassanho Forster

**Affiliations:** 1 Doctoral student, Faculdade de Medicina de Ribeirão Preto, Universidade de São Paulo, Ribeirão Preto, SP, Brazil. Technical consultant, Pan American Health Organization/World Health Organization, Washington, DC, United States of America.; World Health Organization, Pan American Health Organization/World Health Organization, Washington, DC, United States of America; 2 PhD, Adjunct Professor, Faculdade de Enfermagem, Universidade do Estado do Rio Grande do Norte, Pau dos Ferros, RN, Brazil.; 3 PhD, Professor, Escola de Saúde, Faculdade Meridional, Passo Fundo, RS, Brazil.; 4 PhD, Associate Professor, Faculdade de Medicina de Ribeirão Preto, Universidade de São Paulo, Ribeirão Preto, SP, Brazil.

**Keywords:** Primary Health Care, Interprofessional Relations, Patient Care Team, Family Health Strategy, Physicians, Cooperative Behavior

## Abstract

**Objectives:**

to compare the attitudes regarding interprofessional collaboration of health professionals that make up the Family Health Strategy teams participating in the ‘More Doctors’ (*Mais Médicos*) program; and to identify factors associated with attitudes of interprofessional collaboration.

**Method:**

a descriptive, transversal and comparative study developed with 63 health professionals who responded to the Jefferson Scale of Attitudes Toward Interprofessional Collaboration. The data were statistically analyzed.

**Results:**

the sum of the scale items ranged from 88 to 139 points. The analysis of all the Family Health teams indicated statistically significant differences between the scores of the scale and the professional category and between the scores and the education level, suggesting that nurses and professionals with higher education are more inclined towards collaborative practice. The analysis according to the profile of the doctor - Brazilian, Cuban or foreign exchange doctor - found no statistical differences regarding the physicians’ scores, nor in the scores of the components of teams with different profiles.

**Conclusion:**

the profile did not suggest a statistically significant greater or lesser inclination of the doctors or teams toward interprofessional work. This study can support new studies which will contribute to the analysis of inter-professional collaboration and the impact of the *Mais Médicos* program.

## Introduction

Interprofessional collaboration, understood as that in which professionals act in an integrated way by sharing objectives and placing users in the centrality of the process has been widely discussed. It has been pointed out as a premise capable of reorienting the training and health care model and of raising the ability to respond to the health demands of the population, thus strengthening health systems^1^ ^-^ ^3^.

Faced with ever more dynamic and complex health needs marked by the increase of new infectious, environmental and behavioral risks, the importance of interprofessionality, which presupposes the reconciliation of knowledge and practices, as well as the management of different or even opposing views in a permanent process of sharing among different professionals becomes more evident^4^.

In Brazil, this approach assumes a singular importance based on the premise that “the Unified Health System (*SUS*) is interprofessional”^5^, since in being guided by the principles of comprehensiveness, equity and universality, it provides strong structuring bases for education and interprofessional collaboration. These principles have gained strength with the advent of Primary Health Care (PHC) which through the Family Health Strategy (*Estrategia Saude da Família - ESF*) incorporates diverse professions into teams for shared action as a model for restructuring the *SUS*.

However, since its creation, the Family Health Strategy (*ESF*) faces expansion difficulties related to the scarcity of physicians and their unequal distribution, especially in areas of great social vulnerability, since they are an essential professional for composing the teams. This scenario severely jeopardizes the solvability of this care level, which is the preferred gateway of *SUS* ^6^. 

To cope with this situation, the Brazilian government has implemented several policies of coverage extension and internalization of medicine throughout history. More recently, the ‘More Doctors’ program (*Programa Mais Médicos* - PMM) was implemented in 2013, which comprises a set of actions which seek to alleviate the shortage of doctors in PHC, among them the emergency provision of these professionals in the *FHS* ^7^. To this end, more than 18 thousand Brazilian doctors and foreign exchange doctors have been included in multiprofessional teams, guaranteeing assistance to 63 million Brazilians. 

In addition to the numerical conformation of the teams and the *ESF* expansion, it is expected that the context promoted by the *PMM* will have positive repercussions on the dynamics of the work process and in meeting the needs of the population. In this sense, health practices find an important resource to optimize care outcomes ^(^ ^1^ in the principles of collaboration and interprofessionality.

Despite the multiprofessional conformation of the *ESF* teams, obstacles that obstruct collaborative practice are still perceived in the daily work; among them are the individualistic attitude of the professionals in the process of teamwork^8^, which stems from a process of uniprofessional academic training^9^.

Given the short implementation time of the *PMM*, studies that focus on the impact of this program are still scarce, and there are no studies that seek to evidence differences related to aspects of interprofessional work.

Thus, this article assumes the collaborative practice within the scope of the *PMM* as its central theme, with the aim of answering the following research question: 

- Are there differences in attitudes towards interprofessional collaboration of professionals who make up the Family Health Strategy teams participating in the *PMM*? 

Thus, the objectives outlined for this study were: to compare the attitudes toward the interprofessional collaboration of health professionals who make up the Family Health Strategy teams participating in the *PMM*, taking into account three different profiles: one team with Brazilian doctors, a team with foreign exchange doctors, and a team with doctors resulting from the cooperation between the Pan American Health Organization (PAHO), Brazil and Cuba; and to identify factors associated with attitudes towards interprofessional collaboration.

## Method

A descriptive, cross-sectional, comparative and quantitative study developed in Minas Gerais, Brazil, in the territory under the action of four *PMM* supervisory institutions in the state: Central Region - *Secretaria Municipal de Saúde de Belo Horizonte* (*SMSA-BH*); North Region - *Universidade Estadual de Montes Claros* (*Unimontes*); *Vale do Jequitinhonha* - *Universidade Federal dos Vales do Jequitinhonha and Mucuri* (*UFVJM*); and *Triângulo Mineiro* - *Universidade Federal de Uberlândia* (*UFU*).

These institutions are together responsible for supervising 448 physicians in the axis of emergency provision members of family health teams, whose group of professionals correspond to the study population.

The inclusion criterion adopted for the purpose of selecting the physicians were: having completed the first training cycle of the project - the Specialization Course in Family Health.

In turn, health professionals should work directly with the physician, and preferably (but not exclusively) as part of the minimum *ESF* team, with a minimum performance time in the *ESF* of one year. 

The defined exclusion criteria were: professionals on vacations, on leave or removed from their functions during the data collection period.

In order to determine the sample, three *ESF* teams from each activity area (supervisory institution) were randomly included by draw, with one of each profile: one team with Brazilian doctors; a team with foreign exchange doctors; and a team with Cuban doctors. There was an exception applied to the *UFVJM* territory, in which only two teams were included due to the foreign exchange doctor of the drawn team having finalized their contract and returning to their country of origin days prior to data collection. Two new teams were subsequently drawn, although the invitation was not accepted by the doctors. 

The final sample consisted of 63 subjects, including 11 physicians and one social worker who composed one of the *ESF* teams.

The data were collected in November and December of 2016, using the Jefferson Scale of Attitudes Toward Interprofessional Collaboration (JeffSATIC), which aims to measure the attitude of health professionals towards interprofessional collaboration. This instrument was recently transculturally adapted and validated in Brazil. The adaptation followed the steps of translation, back-translation, expert committee evaluation and pre-test application. The instrument was subsequently submitted to construct validation and reliability with 128 PHC professionals^10^. 

The original *Jefferson Scale of Attitudes Toward Interprofessional Collaboration* (JeffSATIC) instrument was developed in 2014^11^, and was tested and validated with 1,976 American and Australian students of different health professions. 

JeffSATIC is structured into 20 items that must be answered using agreement/disagreement variables according to a seven-point Likert scale, in which the lowest level corresponds to *Strongly disagree* (1), and the highest to *Strongly agree* (7). The attitude toward collaboration is reflected by the total score on the scale which can range from 20 to 140, with higher scores indicating more positive attitudes.

A questionnaire was also applied to determine the respondent’s profile, with variables related to their training and work history. 

Data were statistically analyzed using SPSS version 21. A significance of 5% was assumed for the hypothesis tests.

Eight of the 20 items on the scale are inversely scored, and therefore such items were recoded for the analysis using the inversion of the points in an equivalent way, as recommended by the authors^10^ ^-^ ^11^.

The internal consistency of the instrument was tested by Cronbach’s Alpha test, considering a value greater than 0.7 as a good level of consistency. 

The comparisons of the total JeffSATIC scores were made using gross scores: mean, median and standard deviation. The Spearman correlation test was used in order to identify the association degree between the JeffSATIC responses with the training time, professional performance, performance in the *SUS* and in the *ESF*. 

The Mann-Whitney U test was adopted to understand the differences in the distribution of scale response among professionals with different degrees of instruction, and the Kruskal-Wallis test was used to test the difference between the responses according to the professional category and team type. The multiple comparisons Dunn test with Bonferroni correction was applied when the rejection of the null hypothesis occurred in this analysis, with the purpose of identifying which pairs presented statistically significant distribution.

In compliance with the guidelines of Resolution No. 466 of the National Health Council of Brazil of December 12, 2012, the project was submitted to the Research Ethics Committee of the University of São Paulo, under CAAE 47794815.6.0000.5414, and was approved by Opinion No. 1.265.019, dated October 6, 2015.

## Results

The study included 63 professionals, of whom 88.9% were women. The mean age was 38.6 years (± 9.04) (Table 1). 

Considering the answers of the scale by the 63 respondents, the final score of the sum of the items ranged from 88 to 139 points, with a median of 121 (± 11.97; 95% CI: 119.56-122.09). A high level of internal consistency was observed in the scale determined by the Cronbrach alpha of 0.71.

**Table 1:** Sociodemographic profile of professionals in the Family Health Strategy (n=63). Minas Gerais, Brazil, 2016

Variables	Mean (± SD*)	CI^†^ 95% of the mean
Age (years)	38.6 (±9)	[36.3 - 40.8]
Training time (years)	9.78 (±7.9)	[6.65 - 12.90]
Years in profession	8.23 (±6.6)	[6.56 - 9.89]
Years working in the Unified Health System	6.3 (±5.3)	[4.96 - 7.63]
Years in the Family Health Strategy	5.36 (±4.2)	[4.28 - 6.43]
Gender	n	%
Female	56	88.9
Male	7	11.1
Area of activity		
Uberlândia	21	33.3
Belo Horizonte	13	20.6
Montes Carlos	21	33.3
Diamantina/Couto Magalhães	8	12.7
Higher education		
No	35	55.6
Yes	28	44.4
Specialization		
Yes	19	30.2
No	9	14.3
Not applicable	36	55.6
Team Profile		
Brazilian	18	28.6
Cuban	27	42.9
Foreign Exchange	18	28.6
Profession		
Community health agent	31	49.2
Social worker	1	1.6
Nurse	11	17.5
Doctor	11	17.5
Nursing technician/assistant	9	14.3

*SD - Standard deviation; †CI - Confidence interval

The Kruskal-Wallis test was conducted to determine whether there were differences in the JeffSATIC scores of physicians of different profiles, and did not indicate statistically significant differences (p=0.315). The profile of the *PMM* team also did not produce differences in the professionals’ scores (p=0.924). This implies that the doctor profile, whether Brazilian, Cuban or foreign exchange did not reflect in more or less inclination of the team members toward interprofessional work in this sample (Table 2).

As Table 3 shows, the comparison of the scores between the different professional groups resulted in differences with statistical significance (p=0.001), with higher scores for nurses. The post hoc analysis to identify pairs that differed revealed statistically significant differences in the scores between nurses and community agents (p=0.001).

A statistically higher difference was identified among professionals with a higher level (p<0.001), while the score of professionals with specialization did not differ (p=0.383). 

The nationality and legal status of higher education institutions where doctors graduated from did not attribute significant differences in relation to the scores obtained with the responses from these professionals (p=0.662, p=1, in this order), as indicated in Table 4.

The analysis between the mean of the scale responses resulted in a positive and significant correlation with the profession time (R_(s)_= 0368; p=0.003), indicating that the longer the profession time, the greater the willingness towards collaborative attitudes. In turn, a comparative evaluation between the scale score and the training time of the respondents (R_(s)_=-003; p=0.987), performance time in the *SUS* (R_(s)_=-008; p=0.973) and performance time in the ESF (R_(s)_=-030; p=0.897) did not indicate any correlation.

**Table 2:** Analysis of responses to the Jefferson Scale of Attitudes Toward Interprofessional Collaboration of Family Health Strategy professionals and the team’s profile according to the *Mais Médicos* program. Minas Gerais, Brazil, 2016

Team Profile	N	Mean	Median	Standard deviation	p-value
Doctors (n=11)					
Brazilian	4	120.7	121	3.3	0.315
Cuban (recruited)	4	127.2	127.5	4.1	
Foreign Exchange	3	126.6	132	15.6	
Teams (n=63)					
Brazilian	18	119	121	12.2	0.924
Cuban (recruited)	27	119.4	122	12.5	
Foreign Exchange	18	118.5	117.5	11.5	

**Table 3:** Analysis of responses to the Jefferson Scale of Attitudes Toward Interprofessional Collaboration and the different professionals of the Family Health Strategy (n = 63). Minas Gerais, Brazil, 2016

Occupation	N	Mean	Median	Standard deviation	95% confidence interval for the mean		p-value
					Inferior limit	Superior limit	
Community agent	31	112.7	110	11.91	108.3	117	0.001
Social Worker	1	-	-	-	-	-	
Nursing	11	128	130	7.97	122.6	133.3	
Medicine	11	124.7	124	8.22	119.2	130.2	
Nursing assistant / technician	9	122.4	126	9.26	115.3	129.5	

**Table 4:** Analysis of responses to the Jefferson Scale of Attitudes Toward Interprofessional Collaboration of Family Health Strategy professionals and variables related to academic training. Minas Gerais, Brazil, 2016

Variable	N	Mean	Median	Standard deviation	p-value
Team (n=63)					
Higher education					
No	35	113.7	115	11.9	0.000
Yes	28	125.7	127	8.2	
Specialization					
No	9	122.1	129	11.1	0.383
Yes	19	127.4	127	6.1	
Doctors (n=11)					
Nationality					
Brazilian	6	123.8	123.5	5.5	0.662
Foreign	5	125.8	127	11.3	
Nature of the IHE*					
Public	7	124.5	124	9.5	1.00
Private	4	125	125.5	6.4	

*IHE - Institution of Higher Education

## Discussion

This study adds updated knowledge about interprofessional collaboration between different components of *ESF* teams in Brazil, which presupposes mutual effort, dialogue and sharing of information and actions in view of the solvability of the population’s health problems. This implies knowing and understanding the responsibilities of each professional and their importance as a team member from a horizontal perspective^12^. In this understanding, the professional relations built under the historical bias of the hierarchy can affect the attitudes of the health workers in relation to collaborative practice.

In this study, we found that nurses’ attitudes were more positive than those of other professionals. This result is consistent with previous findings from studies that applied similar scales to those used in this research in order to establish comparisons between physicians and nurses^13^ ^-^ ^17^. 

The involvement of the nursing professional is vital for advancing PHC, and consequently of the *SUS* and the expansion of access to primary care based on scientific and safe evidence^18^. Thus, both nurses and physicians need to strengthen collaborative relationships and work side-by-side with the goal of providing effective care to the population^19^.

Despite the highlight of the group of nurses, the mean score of the different professional groups was high (> 112), which shows that all categories have attitudes in favor of interprofessional collaboration, as found in another study^20^.

In this sample, higher education acted as an intervening variable for the scale result, indicating greater availability of undergraduates towards collaboration. A positive relationship between years of professional practice and interprofessional collaboration was also found, which was similar to a study conducted with American physicians and nurses^21^.

Regarding the training in health education, the literature points out that there is still resistance to overcoming training processes that legitimize a healthcare model based on the work fragmentation. Thus, professionals continue to be trained from a uniprofessional perspective to work in a team, a contradiction that has implications for the development and quality of health actions offered^22^. 

In this sense, the development of collaborative skills is still a challenge for the hegemonic health training model, since it focuses on the development of specific skills, strengthening what the literature has called tribalism of the professions^23^ ^-^ ^24^. 

One of the strongest characteristics and which justifies collaboration as a differentiating factor for reorganizing health practices is the user centrality in the production of health services.

It is this centrality that demands other principles of collaboration: sharing common goals; a partnership that encourages a permanent process of interaction; interdependence as recognition of the complementary character of the different professions; and balance of powers between them^25^. 

In this perspective, there is much to be done to ensure that professional training in health is focused on the population and the health systems needs and for health professionals to act in an integrated way in favor of providing comprehensive care as foreseen by *SUS* guidelines^26^.

Operationalizing Health Reform in Brazil has lasted longer than in countries with health systems oriented by PHC such as Canada, Spain and Cuba. In these countries, training has been regulated based on the PHC since the 1980s, assuming medical residency in the area as a gold standard for the training and as a requirement for being incorporated into the health system^27^ ^-^ ^29^. 

In this sense, a study produced in relation to the *PMM* indicates that foreign exchange and recruited doctors possess practical skills and experiences which differ from those of Brazilian physicians, such as centrality in the community approach; the sharing of knowledge and practices with the team as a whole; and the establishment of close bonds with the user and the community. These competences contrast with the practice of most Brazilian physicians, who still maintain excessive centrality in the individual and in a uniprofessional approach^30^. 

Thus, one could expect that the different medical profiles would produce statistical differences in relation to interprofessional attitudes, which was not observed in this study. However, the scores from foreign exchange and recruited doctors were higher than those from Brazilian physicians.

Considering that all the doctors included in this study had completed the first training cycle of the project (Specialization in Family Health), this may have been one of the reasons why no differences were found among the different profiles of physicians. 

It is a fact that the *PMM* is recent and more evidence is still needed regarding the implications of the presence of emergency-deployed doctors in the dynamics of health work, which is, in essence, collective. However, it is believed that the *PMM* constitutes a strong mechanism for an exchange of experiences and practices between foreign and Brazilian professionals, and to produce changes in the work process.

Limitations of the study include determining the sample without calculation, so that the sample size may not be representative. Therefore, generalizing the results should be considered with caution. The lack of studies with documented application of the JeffSATIC can also be pointed out as a limitation, since it restricts data comparison. 

Nevertheless, this study is unprecedented in Brazil, and is the starting point for new research that contributes to deepen the analysis on the interprofessional collaboration in *ESF* teams and to evaluate the impact of the *PMM* under different perspectives.

## Conclusion

The results indicated that a Brazilian, Cuban or foreign exchange profile did not imply in statistical differences related to collaborative attitudes among the studied *PMM* teams; on the other hand, a longer training time and longer professional activity time had a positive effect on collaborative practices. It was also evidenced that the previously demonstrated divergent perspectives in other studies regarding interprofessional collaboration between different professionals may also exist in the PHC, highlighting nurses as the most inclined professional category for collaborative practices.

Future studies are needed to test the influence of different variables on the professional attitude toward collaborative practices comparing interprofessional attitudes between participating and non-participating teams in the *PMM*.
